# Supplementary material for: Predicting Ischemic Stroke Patients to Transfer for Endovascular Thrombectomy Using Machine Learning: A Case Study
Source: Healthcare (Basel). 2025 Jun 16;13(12):1435. doi: 10.3390/healthcare13121435 (PMC12193547; doi:10.3390/healthcare13121435)
Supplement: Supplementary file 1 [file healthcare-13-01435-s001.zip › healthcare-3656795-supplementary.pdf]

Title: Predicting Ischemic Stroke Patients to Transfer for Endovascular Thrombectomy Using Machine Learning: A Case Study

Supplemental Material

Supplemental Figure

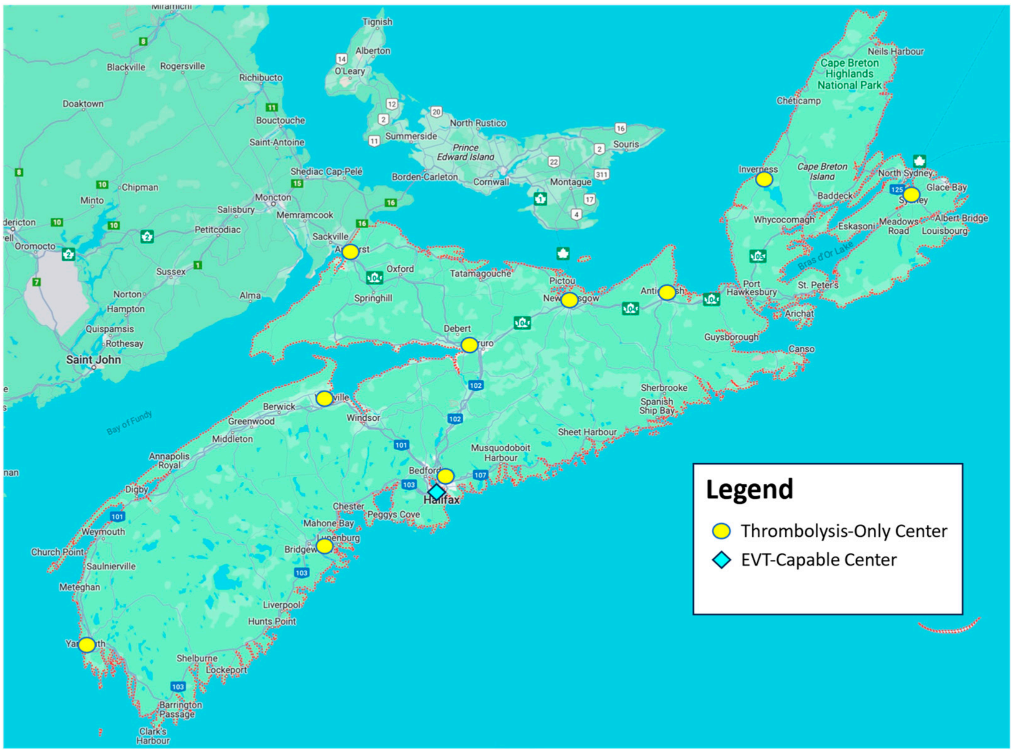

Figure S1. **Location of all ten thrombolysis-only centers and the EVT-capable center in Nova Scotia. EVT: Endovascular Thrombectomy.**

Supplementary Table

Table S1: Hyperparameter summary for each model employed

Model	Hyperparameters
Support Vector Machine	C: 1<br>kernel: linear
Random Forest	n_estimators: 250<br>min_sample_split: 5<br>min_sample_leaf: 2<br>max_features: 6<br>max_depth: 6<br>criterion: gini<br>bootstrap: false
Logistic Regression	C: 10<br>penalty: l2<br>solver: liblinear
Decision Tree	max_depth: 6<br>max_features: 6<br>min_sample_split: 5<br>criterion: gini
Ensemble Model (SVM + RF + LR + DT)	svm_C: 1<br>svm_kernel: rbf<br>rf_n_estimators: 100<br>rf_min_samples_leaf: 1<br>rf_min_samples_split: 2<br>rf_criterion: gini<br>rf_bootstrap: true<br>rf_max_depth: none<br>rf_max_features: sqrt<br>lr_C: 1<br>lr_penalty: l2<br>lr_solver: lbfgs<br>dt_max_depth: none<br>dt_max_features: none<br>dt_min_samples_split: 2<br>dt_min_samples_leaf: 1<br>dt_criterion: gini
